# Supplementary material for: Dendritic Cell-Derived Artificial Microvesicles Inhibit RLS40 Lymphosarcoma Growth in Mice via Stimulation of Th1/Th17 Immune Response
Source: Pharmaceutics. 2022 Nov 21;14(11):2542. doi: 10.3390/pharmaceutics14112542 (PMC9696603; doi:10.3390/pharmaceutics14112542)
Supplement: Supplementary file 1 [file pharmaceutics-14-02542-s001.zip › pharmaceutics-2028666-supplementary.pdf]

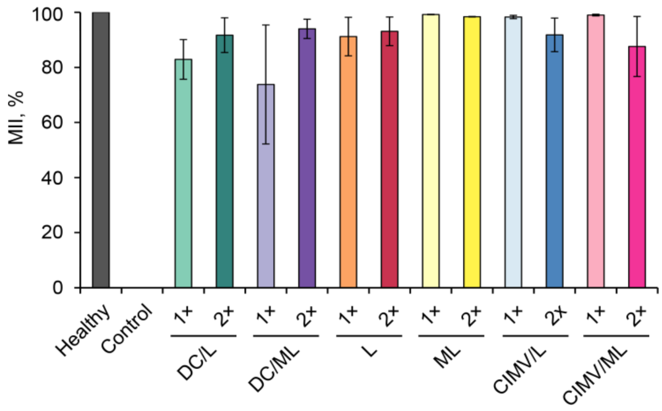

Figure S1. The effect of different types of the vaccines on the liver metastases in RLS_{40}-bearing mice. Metastasis inhibition index (MII) in the control (Opti-MEM) and experimental groups was calculated as MII = ((mean metastasis area$_{control}$ − mean metastasis area$_{experiment}$)/mean metastasis area$_{control}$) × 100%. The data were statistically analyzed using Student's *t*-test and are presented as mean ± S.E.M. Statistical significance: $p \leq 0.05$.

Table S1. Morphometry of RLS_{40} lymphosarcoma tumor tissue of mice without treatment (control) and after vaccines administration.

Groups	Color code	Unchanged tumor tissue, Vv, %	Inflammatory infiltration, Vv, %	Necrotic changes, Vv, %
Control		88.5±1.9	6.2±1.5	4.8±0.5
DC/L(×1)		n.d.*	n.d.	n.d.
DC/L(×2)		71.8±3.6#	21.6±4.2#	6.6±1.4
DC/ML(×1)		n.d.	n.d.	n.d.
DC/ML(×2)		78±2.1#	13.8±1.8#	9±1.5#
L(×1)		n.d.	n.d.	n.d.
L(×2)		82.9±7.6	9.4±5.3	7.8±2.5
ML(×1)		n.d.	n.d.	n.d.
ML(×2)		84.3±6.3	5.5±2.2	10.2±4.1#
CIMV/L(×1)		n.d.	n.d.	n.d.
CIMV/L(×2)		79.6±1.9#	12.5±2.3#	8±2.7
CIMV/ML(×1)		n.d.	n.d.	n.d.
CIMV/ML(×2)		87.3±2	7.9±1.2	5±0.6

* n.d. – not determined. # differences from the control mice were statistically significant at $p \leq 0.05$.

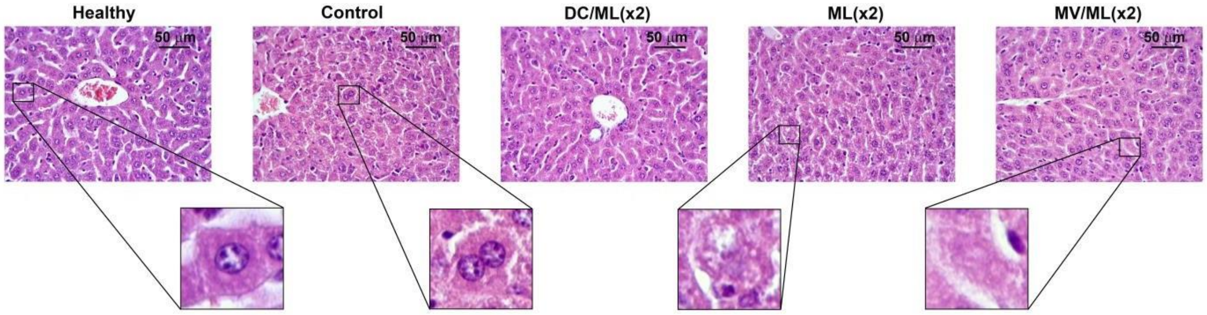

Figure S2. The effect of vaccines on liver in RLS_{40}-bearing mice. Representative histological images of liver sections. Hematoxylin and eosin staining. Black boxes indicate typical examples of normal hepatocyte, binuclear hepatocyte, dystrophy and necrosis. Magnification ×400.

Table S2. The effect of the vaccines on blood biochemistry of RLS$_{40}$-bearing mice.

Groups	Color code	ALT, U/L	AST, U/L	ALK, U/L	Total protein, g/L
Healthy		42±1.6	67.3±1.5	68±5.5	55.4±2.1
Control		41.4±1.7	116.2±3.7*	63.1±2.7	49.9±0.9
DC/L(×1)		45.2±5.9	112±11.1*	90.1±25.8	55.4±4.9
DC/L(×2)		36.9±5.1	85.1±3$^{*\#}$	117.3±33.5$^{*\#}$	55.8±5.4
DC/ML(×1)		39.5±6.7	90.3±5.1$^{*\#}$	97.8±6.9$^{*\#}$	51.5±4.2
DC/ML(×2)		40.5±4.1	89.5±2.1$^{*\#}$	111.5±23.7$^{*\#}$	54.4±2.8$^{\#}$
L(×1)		43.5±2.8	93.6±3.2$^{*\#}$	110.2±23.7$^{*\#}$	56.6±2.7$^{\#}$
L(×2)		38±1.9*	113.6±19.2*	114±22.5$^{*\#}$	54.7±4.1
ML(×1)		28.1±4.2$^{*\#}$	74.1±3.8$^{*\#}$	61.7±6.5	48.5±2.7*
ML(×2)		36±3$^{*\#}$	101.7±5.5$^{*\#}$	100.7±22.8$^{*\#}$	53.5±3.7
CIMV/L(×1)		42.5±5.3	123.4±15.7*	81.4±12.5$^{\#}$	52.4±2.9
CIMV/L(×2)		42.5±5.3	85.5±14.8$^{*\#}$	88.6±22.3$^{\#}$	52.4±2.9
CIMV/ML(×1)		34±3$^{*\#}$	69.7±7.9$^{\#}$	81.7±15.7$^{\#}$	49.3±4.5
CIMV/ML(×2)		49.8±10.2	91.5±3$^{*\#}$	110.5±14.3$^{*\#}$	55.4±3$^{\#}$

* statistically significant difference relative to healthy CBA mice, $p \leq 0.05$ $^{\#}$ statistically significant difference relative to the control RLS$_{40}$-bearing mice, $p \leq 0.05$

Table S3. Liver morphometry of RLS_{40} lymphosarcoma-bearing mice without treatment and after vaccines administration.

Groups	Color code	Normal liver parenchyma, Vv, %	Dystrophy, Vv, %	Necrosis, Vv, %	Total destructive changes, Vv, %	Binuclear hepatocytes, Nv
Healthy		76.9±1.5	7.3±0.7	3.1±0.4	10.4±0.9	1.5±0.2
Control		65.8±2.9*	10.4±1.5*	12.9±1.6*	23.3±3*	1.4±0.2
DC/L(×2)		54.2±2.5*#	18±2.2*#	19.9±1*#	37.9±3*#	1.7±0.2
DC/ML(×2)		65.4±1.6*	8±0.6*	15.3±1.1*	23.3±1.7*	1.7±0.8
L(×2)		58.5±2.7*#	11.8±5.2*	22.3±2.6*#	34.1±3.4*#	1.9±0.3
ML(×2)		62.2±5.4*	12±2.7*	17.8±3.4*	29.8±5.9*	1.4±0.5
CIMV/L(×2)		63.6±2.9*	11.4±2.3*	13.9±1.6*	25.2±3.4*	2.5±1.1
CIMV/ML(×2)		66.3±3.3*	12.6±2*	14.4±1.5*	27±2.8*	4.1±0.9*#

* statistically significant difference relative to healthy CBA mice, $p \leq 0.05$ # statistically significant difference relative to RLS_{40}-bearing mice without treatment (control), $p \leq 0.05$

Table S4. Spleen and thymus morphometry of RLS_{40} lymphosarcoma-bearing mice without treatment (control) and after vaccine administration.

**Groups**	**Color code**	**Spleen morphometry**			**Thymus morphometry**		
		**Red pulp, Vv, %**	**White pulp, Vv, %**	**Diameter of follicles, µm**	**Cortex, Vv, %**	**Medulla, Vv, %**	**C/M index**
Healthy		57.3±3.1	42.6±1.9	229.9±13.5	49.9±1.6	45±1.9	1.1±0.1
Control		55.7±3.5	42.3±3.3	235.5±16.5	50.6±2.3	47.8±2.4	1.1±0.1
DC/L(×1)		44.8±2.5$^{*\#}$	53.9±2.5$^{*\#}$	197.6±22.9	78.4±6.8$^{*\#}$	26.8±1.7$^{*\#}$	3±0.4$^{*\#}$
DC/L(×2)		33.6±2.3$^{*\#}$	61.4±3$^{*\#}$	313.3±21.8$^{*\#}$	72.8±1.9$^{*\#}$	24±2.3$^{*\#}$	3.2±0.4$^{*\#}$
DC/ML(×1)		39.2±4.1$^{*\#}$	60.7±4.2$^{*\#}$	227.6±9.2	73.8±3.8$^{*\#}$	26.2±3.8$^{*\#}$	3±0.6$^{*\#}$
DC/ML(×2)		29±3.1$^{*\#}$	68.8±3.2$^{*\#}$	309.5±12.4$^{*\#}$	68.5±3.4$^{*\#}$	28.1±3.4$^{*\#}$	2.5±0.4$^{*\#}$
L(×1)		60.7±7.6	38.9±7.6	249.5±17.9	55.4±2.6*	43±3.9	1.4±0.3
L(×2)		39±2.3$^{*\#}$	39.1±2.3$^{*\#}$	271.1±9.6$^{*\#}$	65.1±2.9$^{*\#}$	34.3±2.5$^{*\#}$	1.9±0.2$^{*\#}$
ML(×1)		48.6±4.8*	51.8±5.6$^{*\#}$	209±7.2$^{*\#}$	63.2±2.5$^{*\#}$	36.8±2.5$^{*\#}$	1.7±0.2$^{*\#}$
ML(×2)		41.6±1.6$^{*\#}$	54.6±2$^{*\#}$	255±6.3$^{*\#}$	61.4±7.8$^{*\#}$	37.3±8.4$^{*\#}$	1.9±0.5$^{*\#}$
CIMV/L(×1)		46.7±2.9$^{*\#}$	52.9±3.8$^{*\#}$	286±46.3$^{*\#}$	78.6±6.1$^{*\#}$	21.4±.1$^{*\#}$	7.5±3.7$^{*\#}$
CIMV/L(×2)		33.9±4.3$^{*\#}$	62.5±4.1$^{*\#}$	369.8±9.3$^{*\#}$	88.5±2.6$^{*\#}$	9.9±2.7$^{*\#}$	10.3±2.4$^{*\#}$
CIMV/ML(×1)		41.5±4.7$^{*\#}$	58.1±4.8$^{*\#}$	289.7±20.1$^{*\#}$	74.2±3.5$^{*\#}$	25.7±3.6$^{*\#}$	3.3±0.7$^{*\#}$
CIMV/ML(×2)		27±3.9$^{*\#}$	71±4.2$^{*\#}$	321±32.1$^{*\#}$	85.4±5.4$^{*\#}$	13.5±5$^{*\#}$	13±4.6$^{*\#}$

* Statistically significant difference relative to healthy CBA mice, $p \leq 0.05$. # Statistically significant difference relative to RLS_{40}-bearing mice without treatment, $p \leq 0.05$
